# Hidradenitis suppurativa in patients of color is associated with increased disease severity and healthcare utilization: A retrospective analysis of 2 U.S. cohorts

**DOI:** 10.1016/j.jdin.2021.01.007

**Published:** 2021-03-14

**Authors:** James M. Kilgour, Shufeng Li, Kavita Y. Sarin

**Affiliations:** Department of Dermatology, Stanford University, Stanford, California

**Keywords:** chart review, cohort study, ethnicity, ethnic skin, hidradenitis suppurativa, race, skin of color, ANOVA, analysis of variance, HS, hidradenitis suppurativa

## Abstract

**Background:**

Hidradenitis suppurativa (HS) is known to disproportionately affect patients of color; however, there is a paucity of evidence on how its disease profile varies between races and ethnic groups.

**Objective:**

Explore potential race-dependent differences in the disease profile of HS.

**Methods:**

A retrospective analysis was conducted on HS patients at Stanford Hospital and Clinics. Data were compared in terms of demographics, disease severity, and healthcare utilization between races in adults identified to have at least 2 encounters coded for HS. Validation was conducted using Optum's de-identified Clinformatics Data Mart Database of national insurance claims.

**Results:**

Our cohorts consisted of 939 HS patients seen at Stanford and 13,885 HS patients taken from the national dataset. Black and Hispanic patients had greater healthcare utilization compared to White patients. In addition, Hispanic patients at our institution also had significantly increased disease severity compared to their White counterparts (*χ*^2^*P* = .009). Hispanic patients entered tertiary care at an earlier age (Stanford mean: 30.8 years for Hispanics vs 38.7 for Whites; *P* < .001), while Black patients entered later (Stanford mean: 39.6 years).

**Limitations:**

These cohorts may not be representative of the entire HS patient population.

**Conclusion:**

Our findings suggest that patients of color may have greater healthcare utilization and disease severity compared to other groups.

**Capsule Summary**

• Patients of color with Hidradenitis Suppurativa (HS) are often under-reported and under-represented during clinical trials and studies.
• There are important race-dependent differences in the disease profile of HS. Clinicians and researchers need to consider these differences to inform research design and clinical practice.

## Background

Hidradenitis suppurativa (HS) is a chronic inflammatory skin disease characterized by recurrent inflammatory nodules, abscesses, and sinus tracts. It primarily affects skin in the intertriginous areas of the body, rich in apocrine glands.^1^ Estimates of prevalence of the disease vary from 0.05% to 4% in the general population,^2^ but it is known that race significantly affects this prevalence. Garg et al.^3^ conducted a retrospective analysis of 47,690 HS patients in the U.S. and the data showed a standardized prevalence that was 3 times higher in African Americans compared to Whites. Other authors have also reported that African Americans with HS may have a different disease profile, with 1 study in the U.S. reporting differences in Charleston Comorbidity Index scores (2.89 in African Americans compared to 1.79 in Whites),^4^ and a single institution study in the Bronx, New York, revealing a significantly higher Hurley stage of disease in African Americans.^5^ Udechukwu and Fleischer^6^ used databases to characterize outpatient visits for HS, which were maintained by the National Center for Health Statistics between 2005 and 2011, including the National Ambulatory Medical Care Survey and the outpatient portion of the National Hospital Ambulatory Medical Care Survey. They reported a significantly increased risk for outpatient visits in African Americans compared to Whites, with an odds ratio of 2.45. Interestingly, they also reported an even greater risk for Hispanic patients, with an odds ratio of 5.22, compared to non-Hispanics.

There is a paucity of evidence in the existing literature examining HS by race and ethnicity, despite data demonstrating an increased prevalence of HS in Black patients, and a potentially increased disease burden in both Black and Hispanic patients. This paucity of evidence has been highlighted in reviews of cohort studies and clinical trials, which show persistent under-reporting of race and under-representation of patients of color, particularly of Hispanics.^7^^,^^8^ Given the limited race-based data available for HS, we conducted a retrospective analysis of patients seen at our institution using electronic health records and data from a national insurance claims dataset to better understand how the disease profile may vary between races and ethnic groups.

## Methods

Ethical approval was granted by the Stanford University Institutional Review Board (IRB-54175). Using the Stanford Research Repository (STARR), we identified a cohort of adult patients with at least 2 encounters coded using the ICD-9 or 10 code for HS (705.83 or L73.2) who were seen at Stanford Hospital and Clinics between January 2015 to April 2020. Data on patient demographics and all encounters for HS were extracted from the Stanford electronic medical records system, including the retrieval of the full text of clinical notes. R Studio v3.6.1 was used to calculate descriptive statistics, including the mean number of outpatient and emergency room visits and inpatient admissions per patient. Clinical notes were electronically searched for the word “Hurley” and then manually reviewed for the highest recorded Hurley stage. Results were stratified by race and ethnicity into Non-Hispanic Asian, Black, Pacific Islander, Native American, White, and Hispanic categories. Differences in mean values and proportions were tested using analysis of variance (ANOVA) or *χ*^2^ test, as appropriate.

For validation, a second cohort of patients was identified using the Optum's de-identified Clinformatics Data Mart Database, which is a commercial and Medicare Advantage health claims dataset. Patients in the cohort had at least 3 years of continuous enrolment between January 2013 and June 2019, and had at least 2 diagnoses of HS using the ICD-9 or 10 code. Patients were similarly grouped, and the demographics were tabulated and compared using ANOVA or *χ*^2^ tests. The total number of outpatient and emergency room encounters were calculated for each patient, including encounters where the primary diagnosis was HS or a related diagnosis (i.e. cellulitis or abscess with HS as a secondary diagnosis). Analysis of covariance (ANCOVA) was used to test for differences in the mean number of encounters per patient with adjustments made for length of insurance enrolment.

## Results

**Fig 1:**
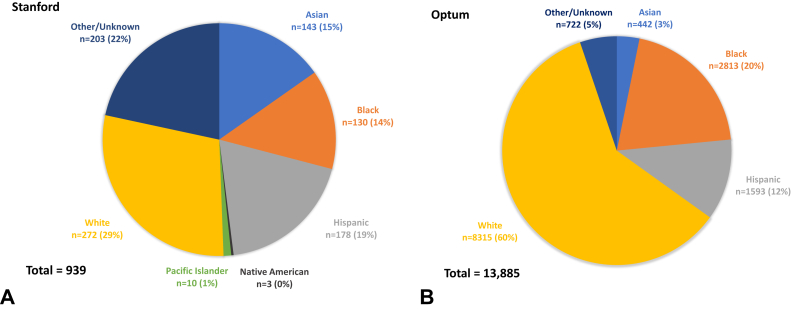
Breakdown of HS Patients by Race in **A**, the Stanford Cohort, and **B**, the Optum Cohort.

The Stanford cohort was composed of 939 HS patients, of which 78% (n = 736) had complete race/ethnicity data. The Optum cohort was composed of 13,885 patients with race/ethnicity data complete for 95% (n = 13,163) of the cohort. The breakdown by group is shown in Fig 1. The Stanford cohort had a greater proportion of Asian (15%; n = 143) and Hispanic patients (19%; n = 178). The Optum cohort had a larger proportion of White (60%; n = 8315) and Black patients (20%; n = 2813). A small number of Native Americans (n = 3) and Pacific Islanders (n = 10) were also identified in the Stanford cohort but not in the Optum cohort (data not presented due to the limited sample size).

In the Stanford cohort, Hispanic patients were placed under tertiary care earlier, with a mean age of first code of 30.8 years, compared to White patients who were placed under tertiary care at a mean age of 38.7, and Black patients with a mean age of 39.6 (Tukey *P* < .001 for both; Table I and Supplementary Table I). At least one Hurley stage was available for 166 patients. Although there was no significant difference in mean stage between groups (ANOVA *P* = .170), Hispanic patients had the highest mean Hurley stage, and a trend toward significance was identified when compared to Whites (mean 2.3 vs 2.1; Tukey *P* = .092; Supplementary Table II). When the proportion of Hurley stages was compared between Hispanics and Whites, the former had a significantly greater proportion of stage 2 and 3 disease compared to the latter (*χ*^2^ *P* = .009; Supplementary Table III). The mean number of outpatient visits was also significantly higher among Hispanics compared to Whites (mean 7.2 vs 4.7; Tukey *P* = .007; Supplementary Table IV). Emergency room visits were additionally increased in Hispanic patients, which trended toward significance compared to Whites (mean 0.2 vs 0.1; Tukey *P* = .092). No significant differences were found in inpatient admissions between the groups (Supplementary Table V, Supplementary Table VI).

**Table I:** Stanford cohort demographics, disease severity, and healthcare utilization

	Asian	Black	Hispanic	White	ANOVA/*χ*^2^ test *P* value
Demographics					
Patients, n (% of total cohort)	143 (15%)	130 (14%)	178 (19%)	272 (29%)	
Gender, n (%)					
Male	54 (37.8%)	27 (20.8%)	48 (27%)	85 (31.3%)	<.001‡
Female	89 (62.2%)	103 (79.2%)	130 (73%)	187 (68.8%)	
Current age mean (SD)	39 (13.7)	44.4 (15.3)	35.1 (11.8)	43.4 (15.7)	<.001‡
Age at presentation mean (SD)	35.1 (13.3)	39.6 (15.2)	30.8 (11.7)	38.7 (15.2)	<.001‡
Disease severity					
Stage I, n (%)	9 (29%)	7 (30.4%)	6 (13%)	22 (35.5%)	
Stage II, n (%)	10 (32.3%)	7 (30.4%)	20 (43.5%)	23 (37.1%)	
Stage III, n (%)	12 (38.7%)	9 (39.1%)	20 (43.5%)	17 (27.4%)	
Mean Hurley stage (SD)	2.10 (0.8)	2.09 (0.9)	2.30 (0.7)	1.92 (0.8)	.170∗
Healthcare utilization—outpatient Visits					
Mean encounters (SD; Range)	4.6 (6.4; 0-47)	5.2 (6; 0-41)	7.2 (10.8; 0-66)	4.7 (5.4; 0-40)	.011†
Healthcare utilization—emergency room visits					
Mean encounters (SD; Range)	0 (0.3; 0-3)	0.2 (0.6; 0-5)	0.2 (1.1; 0-10)	0.1 (0.5; 0-7)	<.001‡
Healthcare utilization—inpatient admissions					
Mean encounters (SD; Range)	0.1 (0.5; 0-3)	0.3 (0.9; 0-5)	0.2 (0.7; 0-7)	0.1 (0.6; 0-5)	<.001‡

Data for Native Americans and Pacific Islanders are not presented here due to small sample size.

*ANOVA*, Analysis of variance; *SD*, standard deviation.

∗ *P* > .05.

† *P* < .05.

‡ *P* < .001.

In the Optum cohort (Table II), Hispanic patients similarly presented earlier to tertiary care alongside Asian patients (mean ages 38.1 and 35.6, respectively), while White and Black patients presented later (means 43.2 and 43.1, respectively; ANOVA *P* < .001; Supplementary Table VII). Black and Hispanic patients tended to be less educated, with 44.9% of Black patients and 37.9% of Hispanic patients possessing only a high school diploma, in contrast to 24.5% of Whites. Furthermore, Black patients were also more impoverished (40.9% had an annual household income less than $40,000 compared to 19.6% of Whites) and had a greater proportion of Medicare coverage (30.6% compared to 23% of Whites). There were significant differences in healthcare utilization in the Optum cohort for both outpatient and emergency room visits (ANCOVA *P =* .004 for both; Supplementary Table VIII, Supplementary Table IX) after controlling for length of insurance program enrolment. Black patients demonstrated the highest healthcare utilization (mean outpatient visits: 5.7 and mean emergency room visits: 0.6). Both mean outpatient visits and mean emergency room visits were significantly higher in Blacks compared to Whites (means 3.6 and 0.3, respectively, ANCOVA *P* < .001 for both). Hispanics also demonstrated a greater mean number of outpatient visits per patient compared to Whites (mean 4.5 vs 3.6) which trended toward significance (ANCOVA *P* = .059), similar to the Stanford cohort.

**Table II:** Optum cohort demographics and healthcare utilization

	Asian	Black	Hispanic	White	ANCOVA/*χ*^2^ test *P* value
Demographics					
Patients, n (% of total cohort)	442 (3%)	2813 (20%)	1593 (12%)	8315 (60%)	
Gender, n (%)					
Male	164 (37.1%)	620 (22%)	409 (25.7%)	2289 (27.5%)	<.001‡
Female	278 (62.9%)	2193 (78%)	1184 (74.3%)	6026 (72.5%)	
Current age mean (SD)	40.3 (14.4)	47.7 (16.7)	42.6 (16.7)	47.7 (17.2)	<.001‡
Age at presentation mean (SD)	35.6 (14.3)	43.1 (16.7)	38.1 (16.7)	43.2 (17.2)	<.001‡
Total years of enrolment Mean (SD)	6.6 (3.4)	6.8 (3.5)	6.9 (3.5)	6.9 (3.4)	.053∗
Education					
Less than 12^th^ grade	<11§	<11§	>30ǁ	15 (0.2%)	<.001‡
High school diploma	60 (13.6%)	1264 (44.9%)	603 (37.9%)	2035 (24.5%)	
Less than bachelor's degree	216 (48.9%)	1391 (49.4%)	789 (49.5%)	4882 (58.7%)	
Bachelor's degree plus	165 (37.3%)	147 (5.2%)	161 (10.1%)	1348 (16.2%)	
Unknown	<11§	<11§	<11§	35 (0.4%)	
Household income					
<$40K	57 (12.9%)	1151 (40.9%)	382 (24%)	1628 (19.6%)	<.001‡
$40K-49K	28 (6.3%)	245 (8.7%)	149 (9.4%)	530 (6.4%)	
$50K-59K	20 (4.5%)	211 (7.5%)	127 (8%)	560 (6.7%)	
$60K-$74K	28 (6.3%)	244 (8.7%)	180 (11.3%)	773 (9.3%)	
$75K-$99K	36 (8.1%)	257 (9.1%)	182 (11.4%)	1167 (14%)	
$100K+	168 (38%)	260 (9.2%)	280 (17.6%)	2424 (29.2%)	
Unknown	105 (23.8%)	445 (15.8%)	293 (18.4%)	1233 (14.8%)	
Insurance type					
Commercial	405 (91.6%)	1951 (69.4%)	1318 (82.7%)	6402 (77%)	<.001‡
Medicare	37 (8.4%)	862 (30.6%)	275 (17.3%)	1913 (23%)	
Healthcare utilization—outpatient visits					
Mean encounters (SD)	4 (10.4)	5.7 (16.8)	4.5 (18.5)	3.6 (9)	.004†
Healthcare utilization—emergency room visits					
Mean encounters (SD)	0.1 (0.5)	0.6 (2.2)	0.4 (1.6)	0.3 (2.1)	.004†

*ANCOVA*, Analysis of covariance; *SD*, standard deviation.

∗ *P* > .05.

† *P* < .01.

‡ *P* < .001.

§ Due to Optum data reporting restrictions, values 10 or under are reported in the table above as “<11.”

ǁ Exact value not reported in order to comply with Optum data reporting restrictions.

## Discussion

Despite the increased prevalence of HS at the population level among patients of color,^3^ the effect of race and ethnicity remains under-investigated. Our study of 2 U.S. cohorts has demonstrated that Hispanic patients tend to receivetertiary care earlier and with increased disease severity. They also have greater levels of healthcare utilization, while Black patients present to tertiary care later but have similarly greater healthcare needs. Further studies are required to elucidate the reasons behind these discrepancies and it is unclear whether these may represent biologic variants or if these differences are the result of the social determinants of health. We noted that Black and Hispanic patients in the Optum cohort tended to have lower levels of education and household incomes, which may lead to healthcare disparities, such as decreased access to more expensive therapies including biologics. HS is known to have a significant economic impact on patients, including slower income growth, increased unemployment, and high indirect costs compared to healthy people,^9^ and this may be further exacerbated by race-dependent structural inequities in society. All patients in the Optum cohort either had commercial insurance or Medicare; therefore, these disparities may be higher in the general population.

Our findings have important implications for research, given the under-representation of patients of color in cohort studies and clinical trials.^7^^,^^8^ It should be a priority for researchers to increase the representation of these patients in designing future trials and a more intensive study of these cohorts is warranted. Our findings also have relevance for clinical practice since HS is known to be a challenging disease to manage and is often marked by an unpredictable disease course.^10^ Previously, obesity and smoking have been identified as important risk factors for disease progression,^11^ and our data adds to this literature by providing evidence that race and ethnicity are potential predictors of increased severity and resource utilization. Race and ethnic background should be considered by dermatologists as parts of a comprehensive and holistic assessment of the patient following the principles of the biopsychosocial model.^12^ Clinicians should also consider the implementation of early interventions for patients of color to slow disease progression and to decrease future healthcare utilization. The findings of our research should also inform education and policy. We advocate that dermatologists should be trained with an awareness of potential race-based disparities in HS patients, and that structural changes should be made within healthcare systems to better address the needs of patients of color. Practical changes could include increasing resource distribution to communities of patients of color, particularly augmenting the availability of specialist HS clinics in areas where emergency room utilization is highest in order to reduce the need for emergency care for HS.

### Limitations

The limitations of this research include the potential skewing of cohort data and the large proportion of patients in the Stanford cohort that had incomplete race and ethnicity data. In addition, only a small subgroup of patients in the Stanford cohort had at least 1 recorded Hurley grade, and no data on disease severity was available in the Optum dataset. Lastly, we used the age at the time of the participant's first code for HS as a surrogate marker for age of presentation to tertiary care. This likely led to an overestimation of the age of disease onset, as it will not capture data from outside of our institution or insurance program. Similarly, patients may have presented to tertiary care at an earlier age, but at that time had a different insurance provider that was not included in our dataset. This discrepancy may disproportionately occur in individuals of lower socioeconomic status due to periods of Medicaid coverage.

## Conclusions

Future research should be conducted to validate our findings within other settings, both in the U.S. and internationally. It is also important to explore the potential underlying physiologic and sociological determinants of HS by race, including barriers to care and potential inequities in terms of access to treatment. Importantly, we call for better representation of patients of color in HS clinical trials and studies, and to attain greater awareness of potential race disparities by clinicians and researchers.

## Conflicts of interest

None disclosed.

## Figures and Tables

**Supplementary Table I:** Tukey testing of differences in mean age at presentation in the Stanford cohort

Groups	Means	ANOVA *P* value	95% confidence interval of difference in means
Asian vs Black	35.1 vs 39.6	.086	−0.4 to 9.3
Asian vs Hispanic	35.1 vs 30.8	.069	−0.2 to 8.8
Asian vs Native American	35.1 vs 36.3	.999	−22.1 to 24.5
Asian vs Pacific Islander	35.1 vs 31.4	.964	−9.3 to 16.8
Asian vs White	35.1 vs 38.7	.133	−0.6 to 7.7
Black vs Hispanic	39.6 vs 30.8	<.001∗	4.2 to 13.4
Black vs Native American	39.6 vs 36.3	.999	−20 to 26.6
Black vs Pacific Islander	39.6 vs 31.4	.470	−4.9 to 21.3
Black vs White	39.6 vs 38.7	.990	−3.3 to 5.2
Hispanic vs Native American	30.8 vs 36.3	.985	−17.8 to 28.7
Hispanic vs Pacific Islander	30.8 vs 31.4	1	−12.4 to 13.5
Hispanic vs White	30.8 vs 38.7	<.001∗	4 to 11.7
Native American vs Pacific Islander	36.3 vs 31.4	.995	−21.4 to 31.2
Native American vs White	36.3 vs 38.7	1	−20.8 to 25.6
Pacific Islander vs White	31.4 vs 38.71	0.582	−5.6 to 20.2

*ANOVA*, Analysis of variance.

∗ *P* < .001.

**Supplementary Table II:** Tukey testing of differences in mean Hurley stage in the Stanford cohort

Groups	Means	ANOVA *P* value	95% confidence interval of difference in means
Asian vs Black	2.1 vs 2.1	1.000	−0.6 to 0.6
Asian vs Hispanic	2.1 vs 2.3	.789	−0.3 to 0.7
Asian vs Pacific Islander	2.1 vs 2.3	.996	−1 to 1.3
Asian vs White	2.1 vs 1.9	.842	−0.3 to 0.7
Black vs Hispanic	2.1 vs 2.3	.816	−0.3 to 0.8
Black vs Pacific Islander	2.1 vs 2.3	.995	−1 to 1.3
Black vs White	2.1 vs 1.9	.906	−0.4 to 0.7
Hispanic vs Pacific Islander	2.3 vs 2.3	1.000	−1.1 to 1.2
Hispanic vs White	2.3 vs 1.9	.092	0 to 0.8
Pacific Islander vs White	2.3 vs 1.9	.925	−0.8 to 1.5

*ANOVA*, Analysis of variance.

**Supplementary Table III:** *χ*^2^ testing of differences in Hurley stage proportions in the Stanford cohort—White versus Hispanic

	Stage I	Stage II	Stage III
Hispanic	6	20	20
White	22	23	17
χ^2^	7.4		
*P* value	.025∗		

	Stage I & II	Stage III
Hispanic	26	20
White	45	17
χ^2^	3	
*P* value	.082	

	Stage I	Stage II & III
Hispanic	6	40
White	22	40
χ^2^	6.9	
*P* value	.009†	

∗ *P* < .05.

† *P* < .01.

**Supplementary Table IV:** Tukey testing of differences in mean number of outpatient visits in the Stanford cohort

Groups	Means	ANOVA *P* value	95% confidence interval of difference in means
Asian vs Black	4.6 vs 5.2	.986	−2 to 3.1
Asian vs Hispanic	4.6 vs 7.2	.020∗	0.3 to 5
Asian vs Native	4.6 vs 7.3	.988	−9.5 to 15
Asian vs Pacific Islander	4.6 vs 5.3	1.000	−6.2 to 7.6
Asian vs White	4.6, 4.7	1.000	−2 to 2.3
Black vs Hispanic	5.2 vs 7.2	.164	−0.4 to 4.5
Black vs Native	5.2 vs 7.3	.996	−10.1 to 14.5
Black vs Pacific Islander	5.2 vs 5.3	1.000	−6.8 to 7
Black vs White	5.2 vs 4.7	.993	−1.8 to 2.7
Hispanic vs Native	7.2 vs 7.3	1.000	−12.1 to 12.4
Hispanic vs Pacific Islander	7.2 vs 5.3	.969	−4.9 to 8.7
Hispanic vs White	7.2 vs 4.7	.007†	0.5 to 4.5
Native vs Pacific Islander	7.3 vs 5.3	.998	−11.8 to 15.9
Native vs White	7.3 vs 4.7	.990	−9.593 to 14.8
Pacific Islander vs White	5.3 vs 4.7	1.000	−6.2 to 7.4

*ANOVA*, Analysis of variance.

∗ *P* < .05.

† *P* < .01.

**Supplementary Table V:** Tukey testing of differences in mean number of emergency room visits in the Stanford cohort

Groups	Means	ANOVA *P* value	95% confidence interval of difference in means
Asian vs Black	0 vs 0.2	.808	−0.1 to 0.3
Asian vs Hispanic	0 vs 0.2	.164	0 to 0.4
Asian vs Native	0 vs 0	1.000	−1.1 to 1.2
Asian vs Pacific Islander	0 vs 0.9	.002†	0.2 to 1.5
Asian vs White	0 vs 0.1	1.000	−0.2 to 0.2
Black vs Hispanic	0.2 vs 0.2	.919	−0.2 to 0.3
Black vs Native	0.2 vs 0	.999	−1 to 1.3
Black vs Pacific Islander	0.2 vs 0.9	.011∗	0.1 to 1.4
Black vs White	0.2 vs 0.1	.786	−0.1 to 0.3
Hispanic vs Native	0.2 vs 0	.993	−0.9 to 1.4
Hispanic vs Pacific Islander	0.2 vs 0.9	.029∗	0 to 1.3
Hispanic vs White	0.2 vs 0.1	.092	0 to 0.4
Native vs Pacific Islander	0 vs 0.9	.343	−0.4 to 2.2
Native vs White	0 vs 0.1	1.000	−1.1 to 1.2
Pacific Islander vs White	0.9 vs 0.1	.002†	0.2 to 1.5

*ANOVA*, Analysis of variance.

∗ *P* < .05.

† *P* < .01.

**Supplementary Table VI:** Tukey testing of differences in mean number of inpatient admissions in the Stanford cohort

Groups	Means	ANOVA *P* value	95% confidence interval of difference in means
Asian vs Black	0.1 vs 0.3	.260	−0.1 to 0.5
Asian vs Hispanic	0.1 vs 0.2	.984	−0.2 to 0.3
Asian vs Native	0.1 vs 1	.344	−0.4 to 2.2
Asian vs Pacific Islander	0.1 vs 1.6	<.001∗	0.8 to 2.2
Asian vs White	0.1 vs 0.1	.998	−0.2 to 0.3
Black vs Hispanic	0.3 vs 0.2	.600	−0.1 to 0.4
Black vs Native	0.3 vs 1	.633	−0.6 to 2
Black vs Pacific Islander	0.3 vs 1.6	<.001∗	0.6 to 2
Black vs White	0.3 vs 0.1	.325	−0.1 to 0.4
Hispanic vs Native	0.2 vs 1	.421	−0.5 to 2.1
Hispanic vs Pacific Islander	0.2 vs 1.6	<.001∗	0.7 to 2.2
Hispanic vs White	0.2 vs 0.1	.999	−0.2 to 0.2
Native vs Pacific Islander	1 vs 1.6	.847	−0.9 to 2.1
Native vs White	1 vs 0.1	.384	−0.4 to 2.2
Pacific Islander vs White	1.6 vs 0.1	<.001∗	0.8 to 2.2

*ANOVA*, Analysis of variance.

∗ *P* < .001.

**Supplementary Table VII:** Tukey testing of differences in mean age at presentation in the Optum cohort

Groups	Means	ANOVA *P* value	95% confidence interval of difference in means
Black vs Asian	43.1 vs 35.6	<.001†	5.3 to 9.7
Hispanic vs Asian	38.1 vs 35.6	.032∗	0.2 to 4.8
White vs Asian	43.2 vs 35.6	<.001†	5.5 to 9.7
Hispanic vs Black	38.1 vs 43.1	<.001†	−6.4 to 3.7
White vs Black	43.2 vs 43.1	.998	−0.9 to 1
White vs Hispanic	43.2 vs 38.1	<.001†	3.9 to 6.3

*ANOVA*, Analysis of variance.

∗ *P* < .05.

† *P* < .001.

**Supplementary Table VIII:** ANCOVA pairwise testing of differences in mean number of outpatient visits, adjusted for years of enrolment, in the Optum cohort

Groups	ANCOVA *P* value
Asian vs Black	.051
Asian vs Hispanic	1.000
Asian vs White	1.000
Black vs Hispanic	.026∗
Black vs White	<.001†
Hispanic vs White	.059

Groups	Adjusted mean	95% confidence interval of adjusted mean
Asian	4	2.8-5.1
Black	5.6	5.2-6.1
Hispanic	4.5	3.9-5.1
White	3.6	3.4-3.9

*ANOVA*, Analysis of variance.

∗ *P* < .05.

† *P* < .001.

**Supplementary Table IX:** ANCOVA pairwise testing of differences in mean number of emergency room visits, adjusted for years of enrolment, in the Optum cohort

Groups	ANCOVA *P* value
Asian vs Black	<.001†
Asian vs Hispanic	.142
Asian vs White	.332
Black vs Hispanic	.001∗
Black vs White	<.001†
Hispanic vs White	1.000

Group	Adjusted mean	95% confidence interval of adjusted mean
Asian	0.1	−0.1 to 0.3
Black	0.6	0.5 to 0.7
Hispanic	0.4	0.3 to 0.5
White	0.3	0.3 to 0.4

ANCOVA, Analysis of covariance.

∗ *P* < .01.

† *P* < .001.
