# Correction

**DOI:** 10.1016/j.jdin.2021.03.006

**Published:** 2021-04-13

**Kilgour JM, Li S, Sarin KY. Hidradenitis suppurativa in patients of color is associated with increased disease severity and healthcare utilization: A retrospective analysis of 2 U.S. cohorts. *JAAD Int*. 2021;3:42-52.**

The publisher regrets that incorrect labels were published on Fig 1. The figure has been replaced online.

